# The *RICE MINUTE-LIKE1* (*RML1*) gene, encoding a ribosomal large subunit protein L3B, regulates leaf morphology and plant architecture in rice

**DOI:** 10.1093/jxb/erw167

**Published:** 2016-05-28

**Authors:** Ming Zheng, Yihua Wang, Xi Liu, Juan Sun, Yunlong Wang, Yang Xu, Jia Lv, Wuhua Long, Xiaopin Zhu, Xiuping Guo, Ling Jiang, Chunming Wang, Jianmin Wan

**Affiliations:** ^1^State Key Laboratory for Crop Genetics and Germplasm Enhancement, Nanjing Agricultural University, Nanjing 210095, P.R. China; ^2^National Key Facility for Crop Gene Resources and Genetic Improvement, Institute of Crop Science, Chinese Academy of Agricultural Sciences, Beijing 100081, P.R. China

**Keywords:** *Minute* mutants, *Oryza sativa*, plant development, plant growth, ribosomal proteins, ribosome biogenesis, rice, vascular patterning.

## Abstract

Mutations of ribosomal proteins (RPs) are known to cause developmental abnormalities in yeast, mammals, and dicotyledonous plants; however, their effects have not been studied in rice. Here, we identifiy a ribosomal biogenesis mutant, *rice minute-like1* (*rml1*) that displays a *minute* phenotype as evidenced by retarded growth and defects in the vascular system. We determine that *RML1* encodes a ribosome large subunit protein 3B (RPL3B) in rice by means of map-based cloning and genetic complementation. *RPL3B* is abundantly expressed in all the tissues, whereas *RPL3A*, another *RPL3* gene family member, is expressed at low levels. Notably, the expression level of *RPL3A* in the *rml1* mutant is similar to that in the wild-type, suggesting that *RPL3A* provides no functional compensation for *RPL3B* in *rml1* plants. Ribosomal profiles show that mutation of *RPL3B* leads to a significant reduction in free 60S ribosomal subunits and polysomes, indicating a ribosomal insufficiency in the *rml1* mutant. Our results demonstrate that the ribosomal protein gene *RPL3B* is required for maintaining normal leaf morphology and plant architecture in rice through its regulation of ribosome biogenesis.

## Highlight

A rice ribosome large subunit protein 3B gene is identified, mutation of which causes abnormal plant architecture. This is the first characterization of a ribosomal protein involved in monocot plant development.

## Introduction

Ribosomes, machines for protein synthesis, are composed of small (40S) and large (60S) subunits in eukaryotes. The small subunit consists of ~33 ribosomal proteins (designated RPS) and 18S rRNA, whereas the large subunit consists of ~47 ribosomal proteins (designated RPL) and 25–28S, 5.8S, and 5S rRNAs (McIntosh and Bonham-Smith, 2006). The genes for most ribosomal proteins (RPs) appear to be evolutionarily conserved among species, such as those of the Archaea, Bacteria, and Eukarya (Lecompte *et al.*, 2002; Mears *et al.*, 2002). In yeast (*Saccharomyces cerevisiae*), two-thirds of the RPs are duplicated (Deutschbauer *et al.*, 2005; Komili *et al.*, 2007; Dean *et al.*, 2008). Most RP families in mammalian species are composed of single-expressed genes and multiple pseudo-copies (Dudov and Perry, 1984). In contrast to mammals, Arabidopsis (*Arabidopsis thaliana*) RPs are involved in development and consist of two to seven copies, making them quite difficult to characterize in detail (Barakat *et al.*, 2001; Blanc and Wolfe, 2004; Thomas *et al.*, 2006).

Previous studies have shown that mutations in ribosomal proteins generally cause deleterious effects on growth and development of an organism (Horiguchi *et al.*, 2012). RP mutations in *Drosophila melanogaster* cause *minute* phenotypes that include delayed larval development, pleiotropic morphological aberrations, smaller body size, and recessive embryo lethality (Kongsuwa *et al.*, 1985; Lambertsson, 1998; Marygold *et al.*, 2007). Growth defects of most *minute* mutants are attributed to decreased levels of ribosomes, which might perturb the translation of specific targets or result in a reduced capacity for global protein synthesis in *Drosophila* development (Marygold *et al.*, 2007).

Mutations of RPs of both small and large subunits in Arabidopsis also exhibit multiple abnormalities, including vascular pattern defects, embryo lethality, retarded root growth, late flowering, or reduced plant size (Byrne, 2009). Some of these (*rps6*, *rps11*, *rpl2*, *rpl8*, *rpl23*, *rpl19*, and *rpl40*) are related to embryo-defective abnormalities (Tzafrir *et al.*, 2004; Meinke *et al.*, 2008). Other RP mutations affect leaf development, such as *pfl1* (*s18a*), *pfl2* (*s13b*), *ae5* (*l28a*), o*l17* (*l5b*), *pgy1* (*l10a*), *pgy2* (*l9*), *pgy3* (*l5a*), and *rpl4a/d* (Van Lijsebettens *et al.*, 1994; Ito *et al.*, 2000; Nishimura *et al.*, 2005; Pinon *et al.*, 2008; Yao *et al.*, 2008; Fujikura *et al.*, 2009). The capacity of protein synthesis in these mutants might be sufficiently decreased to retard cell division or might involve genes that affect auxin distribution in the developing leaves (Scarpella *et al.*, 2006; Byrne, 2009). *PGY* genes are thought to be involved in ribosome-mediated translational regulation of genes in the HD-ZIPIII-KANADI pathway (Pinon *et al.*, 2008; Yao *et al.*, 2008). Similar defects were found to be present in the recently described *short valve1* (*stv1*)/*rpl24* mutant, with cotyledon and leaf vascular patterning defects (Nishimura *et al.*, 2005). In addition, this mutant displayed variable apical–basal gynoecium patterning defects. Recent studies of *stv1*/*rpl24*, *rpl4d*, *rpl5a*, and elongation factor *eif3h* mutants have found that they are involved in the auxin-signaling pathway through an uORF-dependent mechanism by perturbing translation reinitiation of *AUXIN RESPONSE FACTOR* (*ARF*) transcripts, such as *ETTIN* (*ETT*)/*ARF3* and *MONOPTEROS* (*MP*)/*ARF5* (Nishimura *et al.*, 2005; Zhou *et al.*, 2010; Horiguchi *et al.*, 2012; Rosado *et al.*, 2012).

RIBOSOMAL PROTEIN L3 (RPL3) is a highly conserved protein across yeast, animals and plants. In yeast, RPL3 is an essential and indispensable component for the formation of a peptidyltransferase centre (PTC) (Schulze and Nierhaus, 1982). Depletion of RPL3 *in vivo* arrests early assembly of the 60S ribosomal subunits and impairs nucleocytoplasmic export of pre-60S ribosomal particles. Additionally, RPL3-depleted cells are arrested in the G1 phase (Rosado *et al.*, 2007). Several studies have shown that mutant forms of RPL3 have altered ribosome structures, reduced ribosomal peptidyltransferase activity, and decreased rates of cell growth and protein synthesis (Petrov *et al.*, 2004; Meskauskas and Dinman, 2007). Mutations in the ‘W finger’ of RPL3 also affect the structure of 25S rRNA and maturation of pre-40S (Meskauskas and Dinman, 2007; Garcia-Gomez *et al.*, 2014). Mutation of RPL3 in *E. coli* also increases resistance to the peptidyltransferase inhibitor tiamulin by alteration of the binding site for the drug (Bosling *et al.*, 2003; Klitgaard *et al.*, 2015). In Arabidopsis, T-DNA insertion of *RPL3A* results in embryo lethality (Tzafrir *et al.*, 2004). In *Nicotiana tabacum*, *RPL3* has been shown to positively regulate cell division, and silencing of *RPL3* led to retarded development, inhibition of lateral root growth, and a decrease in accumulation of pre-rRNA (Popescu and Tumer, 2004). These studies suggest that the RPL3 also has a regulatory role in plant development.

Despite the characterization of several ribosomal genes that function in yeast and Arabidopsis, mutations of such genes have not yet been identified in rice. In this study, we characterize a *rice minute-like1* mutant (*rml1*), which displays retarded growth, as evidenced by reduced plant height, narrow leaves, inhibited lateral root growth, reduced seed size, and delayed flowering. We show that the *RML1* encodes ribosome large subunit protein L3B (RPL3B). In addition, there is a second copy, *RPL3A*, in the rice genome. We demonstrate that *RPL3A* and *RPL3B* have different expression profiles and functions. We suggest that ribosome aberrancy or polysome insufficiency might be responsible for the aberrant growth and development in the *rml1* mutant. This work enhances our understanding of the regulatory roles of ribosomal genes in plant development.

## Materials and methods

### Plant materials and growth conditions

The *rml1* mutant was obtained from a ^60^Co-irradiated population of *Oryza sativa indica* rice cv. 93-11. Genetic analysis showed that the mutant phenotype was controlled by one recessive gene (see Supplementary Fig. S1 at *JXB* online). Plants were grown in a paddy field at Nanjing Agricultural University, China.

### Histological analysis of leaves and stems

To analyse leaf vasculature, *rml1* and wild-type flag leaves at the mature stage were fixed in FAA (formalin–acetic acid–alcohol) solution, and samples were treated as described by Zheng *et al.* (2015). Embedded tissues were sectioned at 8 μm thickness and stained with 0.05% Toluidine Blue. Whole-mount clearing of *rml1* and wild-type flag leaves was performed as described by Qi *et al.* (2008). Images were observed with a Nikon ECLIPSE80i light microscope. For analysis of cell morphology of the stem, internode I of wild-type and *rml1* were sectioned by a vibratome at 100 μm and stained with a mixture of Calcofluor White (Sigma) and 10% KOH. Microscopic examinations were made under UV light.

### Map-based cloning and complementation test for *RML1*

To identify and map the *RML1* gene, *rml1* was crossed with 02428, a *japonica* cultivar. Ten individuals showing the recessive mutant phenotype were identified in the *rml1*/02428 F_2_ population for preliminary mapping. A further 1200 F_2_ plants with the mutant phenotype were used for fine mapping with SSR/InDel markers designed by comparison of the genomic sequences of Nipponbare (*japonica*) and cv. 93-11.

For complementation of the *rml1* mutation, the wild-type *RML1* genomic DNA sequence was cloned into the binary vector pCAMBIA1300 under its native promoter to generate the binary vector *pRML1*-*gRML1*. Due to ongoing difficulties in performing transformation of the *rml1* mutant allele in the *indica* background, the allele was back-crossed to the *japonica* cv. Dian Jing You (DJY) to derive a line named *r-3*. Plasmid *pRML1*-*gRML1* was introduced into calli of *r-3* by Agrobacterium-mediated transformation as described previously (Hiei and Komari, *et al.*, 2008). To determine whether the *RPL3A* transcript can rescue the mutant phenotype, the full-length *RPL3A* cDNA was cloned into the binary vector under control of the *RPL3B* promoter. Plasmid *pRPL3B*-*cRPL3A* was also transformed into *r-3* calli.

### RNA extraction and real-time RT-PCR analysis

Total RNA was extracted using a RNA Prep Pure Plant Kit (TIANGEN, Beijing) and cDNA was synthesized with Oligo (dT) 18 or a random primer, and reverse-transcribed using PrimeScript Reverse Transcriptase (TaKaRa Bio Inc., Dalian). Real-time RT-PCR was performed using a SYBRGreen Mix Kit (Bio-Rad, Hercules, CA) on an ABI 7500 real-time PCR system with three biological replicates. The rice *ubiquitin* gene *LOC_Os03g13170* was used as an endogenous control. Primers for real-time RT-PCR are listed in Supplementary Table S4. The 2^−ΔΔCT^ method was adopted to analyse relative gene expression (Livak and Schmittgen, 2001).

### Subcellular localization of RML1 protein

The cDNA of *RML1* was amplified from the wild-type and fused with green fluorescent protein (GFP) to generate a pCAMBIA1305 vector. The fusion protein was transiently expressed in epidermal cells of *Nicotiana benthamiana* leaves (primer sequences are listed in Supplementary Table S3). GFP alone was used as the control, and FIB2-mCherry was used as a nuclear marker (Degenhardt and Bonham-Smith, 2008*a*). After 18 or 48h transformation, GFP signals were observed with a confocal laser scanning microscope (Carl Zeiss LSM780).

### *RML1pro:GUS* reporter gene construction and analysis

A 1.8-kb fragment upstream of the *RML1* translation start site was cloned into binary vector pCAMBIA1381Z to fuse with the *GUS* reporter gene (primer sequences are listed in Supplementary Table S3) and transformed into the Nipponbare cultivar by the *Agrobacterium*-mediated method. Hygromycin-resistant calli were regenerated and eight positive homozygous lines were obtained. GUS staining was performed as described by Jefferson *et al.* (1987).

### Ribosome profile analysis

Ribosomes were isolated from rice leaves as described previously (Mustroph *et al.*, 2009; Rivera *et al.*, 2015) with minor modifications. Tissue (20g) from 1-week-old seedlings was pulverized in liquid nitrogen and homogenized in 50ml of plant extraction buffer [50mM Tris-HCl (pH 7.5), 400mM KCl, 30mM MgCl_2_, 5mM dithiothreitol, 50mg ml^–1^ cycloheximide, 50mg ml^–1^ chloramphenicol, 1% Triton X-100]. Cell debris was removed by centrifugation at 1076 *g* for 7min at 4 °C. A 0.1 volume of 20% Triton X-100 was added to the supernatant and centrifuged at 17210 *g* for 20min at 4 °C. The supernatant was then loaded onto a 13-ml sucrose cushion [20mM Tris-HCl (pH 7.6), 5mM MgCl_2_, 50mM NH_4_Cl, 60% (w/w) sucrose] and centrifuged at 117000 *g* for 18h at 4 °C. Pellets were re-suspended in 200 μl of cold re-suspension buffer [200mM Tris-HCl (pH 9.0), 200mM KCl, 25mM EGTA, 35mM MgCl_2_, 5mM DTT, 50mg ml^–1^ cycloheximide, and 50mg ml^–1^ chloramphenicol] and incubated at 4 °C for 1h. The ribosomes were layered onto an 11-ml 5–55% linear sucrose gradient and centrifuged at 170000 *g* for 2.5h at 4 °C, and the absorbance of each fraction was measured at 254nm with a fraction collection system (Biocomp). Proteins in each fraction were precipitated in two volumes of ice-chilled ethanol at 4 °C for about 12h.

### Western blotting

Total proteins were extracted from wild-type and *rml1* leaves with buffer B [50mM Tris-HCl, pH 7.5, 150mM NaCl, 0.5% Triton X-100, and protease inhibitor cocktail Complete Mini tablets pH 8.0 (Roche)] (Willige *et al.*, 2011), and boiled in 1× SDS loading buffer at 95 °C for 5min. Proteins were separated in 8–12% SDS-PAGE gradient gels and transferred to polyvinylidene difluoride (PVDF) membranes (0.45 μm, Bio-Rad). Western blots were detected with anti-OsRPL3 (Abmart), anti-hsRPS14 (Millipore), anti-α-tubulin (Sigma) antibodies, and related secondary antibodies, respectively. An ECL reagent (Bio-Rad) was used for imaging.

## Results

### Multiple developmental defects in the *rml1* mutant

**Fig. 1.**
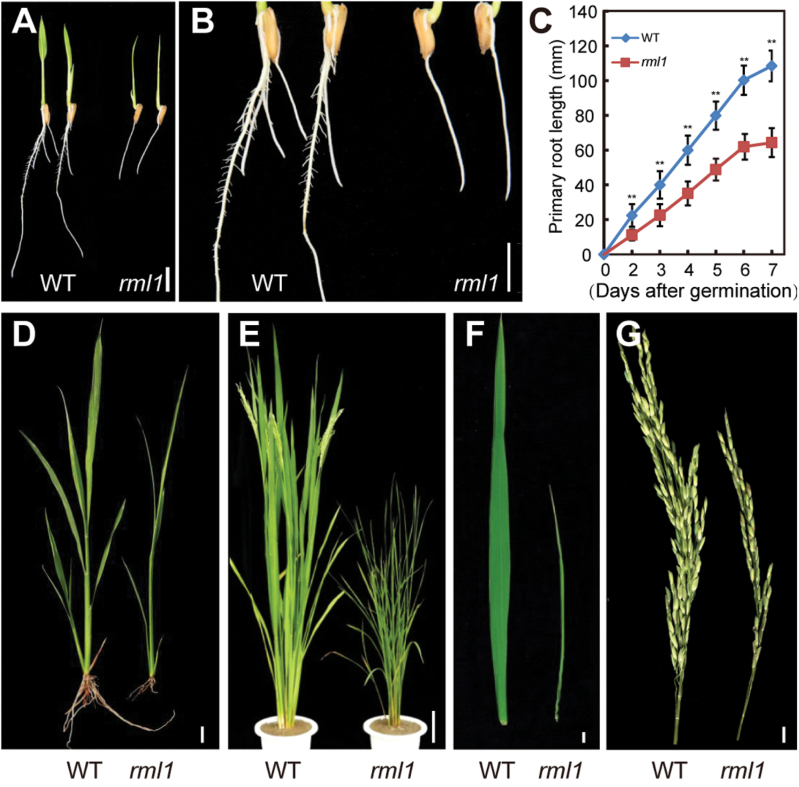
Gross morphology analyses of the wild-type (WT) and *rml1* mutant. (A) Root and shoot phenotypes of 2-d-old wild-type and *rml1* mutant plants. (B) Magnification of wild-type and *rml1* mutant roots in (A). (C) Comparison of primary root growth after germination between wild-type and *rml1* mutants (*n*=20). (D) Phenotypes of the wild-type and the *rml1* mutant at the five-leaf stage when grown in the field. (E) Morphologies of the wild-type and *rml1* mutant plants at heading. (F) Flag leaves of wild-type and *rml1* plants at the heading stage. (G) Main panicles of the wild-type and *rml1* mutant plants. Scale bars: 10mm (A, B, D, F, G); 10cm (E). Student’s *t*-test was used for statistical analysis (*, *P*<0.05; **, *P*<0.01).

We screened a ^60^Co-irradiated population of *indica* rice variety 93-11 in a search for leaf morphology and plant architectural variants. We identified a *minute* mutant that displayed retarded plant growth and development, and named it *rice minute-like 1* (*rml1*). The *rml1* mutant showed delayed seed germination and inhibition of root growth (Fig. 1A, B; Supplementary Fig. S2A). We performed a time-course comparison of primary root lengths of wild-type and *rml1* mutant over days 1–7 following seed germination And found that the wild-type grew faster than *rml1* (Fig. 1C). Wild-type seedlings at the 5th leaf stage in the field were already forming new tillers, whereas the *rml1* mutant was not (Fig. 1D; Supplementary Fig. S2B). At the mature stage, the *rml1* plants had reduced size compared to the wild-type, including dwarfing, narrow leaves, and short panicles (Fig. 1E–G; Table 1). The mutant was ~40 d later flowering than the wild-type (Fig. 1E). Thus, the *rml1* mutant clearly displayed retarded and delayed plant growth relative to its wild-type.

**Table 1.** Morphological traits of wild-type and *rml1* plants

	Tillers per plant	Plant height (cm)	Main panicle length (cm)	Primary branch number per main panicle	Number of spikelets per panicle
Wild-type	9.9±2.6	116.1±4.3	25.0±2.0	11.9±1.1	218.7±13.0
*rml1*	7.4±1.6*	84.0±5.7**	20.3±1.2**	8.2±0.9**	88.0±11.8**

Error bars indicate ±SD (*n*=10). Student’s *t*-test was used for statistical analysis (*, *P*<0.05; **, *P*<0.01).

### Abnormal vascular numbers and defects in *rml1* leaves

**Fig. 2.**
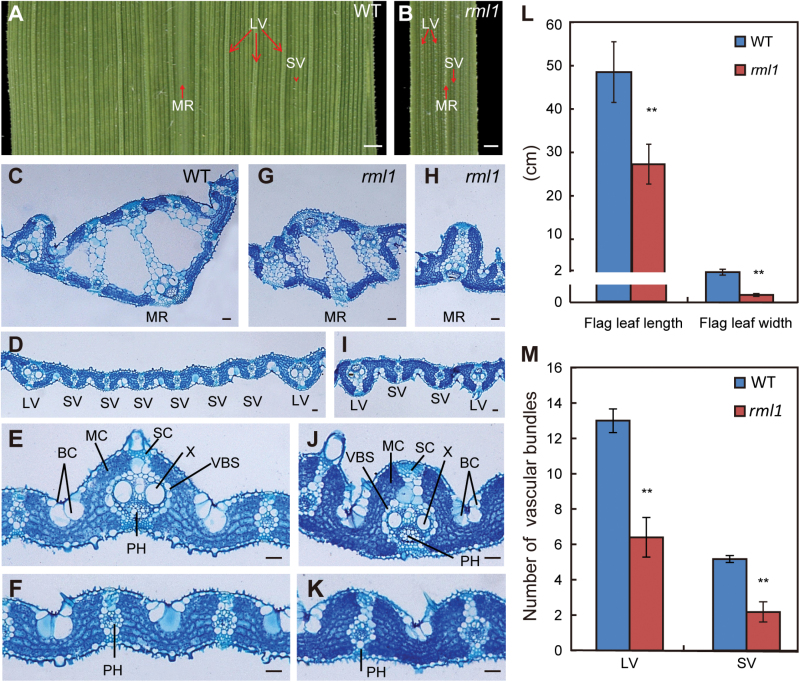
Patterning of vascular bundles in leaves of wild-type (WT) and *rml1* plants. (A, B) Enlarged views of adaxial surfaces of mature flag leaf blades in wild-type and *rml1* plants. (C–K) Transverse sections of the middle part of flag leaf blades in the wild-type and *rml1* plants stained with toluidine blue. (C–F) Magnifications of midrib (C), large vascular bundles (E), and small vascular bundles (F) in the wild-type. (G–K) Magnifications of midrib (G,H) and vascular bundles (I–K) in the *rml1* mutants. (L) Length and width of wild-type and *rml1* flag leaves at maturity (*n*=15). (M) Numbers of large and small vascular bundles (between the two adjacent LVs) in wild-type and *rml1* flag leaves at maturity (*n*=10). Abbreviations: BC, bulliform cells; LV, large vascular bundle; MC, mesophyll cell; MR, midrib; PH, phloem; SC, sclerenchymatous tissue or cell; SV, small vascular bundle; VBS, vascular bundle sheath; X, xylem. Scale bars: 1mm (A, B); 50 μm (C–K). Student’s *t*-test was used for statistical analysis (*, *P*<0.05; **, *P*<0.01).

One of the obvious phenotypes of *rml1* mutant was leaf blade morphology. Leaf blade width in the *rml1* mutant was consistently narrower than in the wild-type from the 5th leaf stage to maturity (Fig. 2A, B). Some leaf blades showed irregular edges and twisted basal midribs (see Supplementary Fig. S3A-a–c). The lengths and widths of mature flag leaves of *rml1* were about 56% and 17% of the wild-type, respectively (Fig. 2L), and sometimes the flag leaves formed a curled structure (Supplementary Fig. S3A-d). Histological observations showed that the number of large veins (LVs) in the leaf blades was significantly reduced in *rml1* flag leaves. Moreover, the number of small veins (SVs) between adjacent LVs was also reduced to less than one-half of the wild-type (Fig. 2A, B; Supplementary Fig. S3B).

We performed transverse sections of mature flag leaf blades in order to characterize the arrangements of vascular bundles in detail. The midrib of the wild-type leaf consisted of several air cavities and vascular bundles (VBs) (Fig. 2C). In contrast, development of midrib in *rml1* mutant was suppressed and sometimes irregular (Fig. 2G, H). The leaf blade of the wild-type had ~13 LVs, with five to six SVs between adjacent LVs (Fig. 2D, Supplementary Fig. S3C). In contrast, the number of LVs in *rml1* was greatly reduced to about half of that in the wild-type. Remarkably, the number of SVs between adjacent LVs was reduced to only one or two (Fig. 2I, M; Supplementary Fig. S3D). *rml1* plants also had abnormal vascular patterning in which the sizes of xylem (X) and bulliform cells (BCs) were also reduced (Fig. 2E, F, J, K). At the edge of the leaf blades, the morphology of VBs was asymmetric (see Supplementary Fig. S3E, F). Other tissues had no noticeable differences except that the size of mesophyll cells in the mutant was slightly smaller than those of the wild-type (Fig. 2; Supplementary Fig. S3). These results indicated that *RML1* controls vascular number and patterning during leaf development.

### *rml1* displays semi-dwarfness

**Fig. 3.**
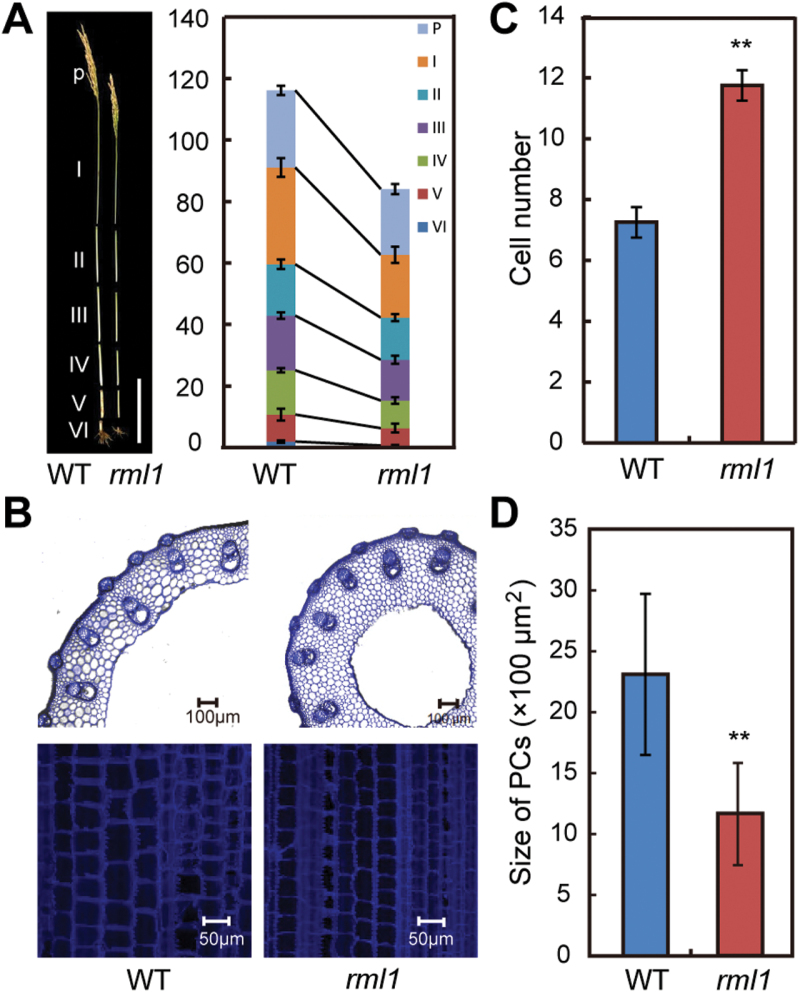
Morphological comparison of wild-type and *rml1* plant height. (A) Phenotypic characterization of internodes of wild-type and *rml1* plants at maturity. P, panicle. I to VI indicate corresponding internodes from top to bottom. (B) Transverse (top) and longitudinal (bottom) sections of the first internode (I) of wild-type and *rml1* plants at maturity. (C) Comparison of parenchyma cell numbers in the transverse sections of the first internode of wild-type and *rml1* plants. (D) Comparison of parenchyma cell (PC) size in longitudinal sections of the first internode of wild-type and *rml1* plants. Student’s *t*-test was used for statistical analysis (*, *P*<0.05; **, *P*<0.01). Scale bar in (A) is 20cm.

In addition to narrow leaves, *rml1* plants exhibited a semi-dwarf stature. The height of *rml1* plants at maturity was about 72% of wild-type, and every internode, including that of the panicle, of *rml1* was significantly shorter (Fig. 3A; Table 1). Examination of cross-sections revealed that the size and number of vascular bundles in the first internode of *rml1* were reduced (Fig. 3B). The cross-sectional width did not differ, but the number of parenchyma cells (PCs) was significantly increased (Fig. 3B, C). In addition, longitudinal sections of the first internode showed that the size of PCs was reduced, but the number was higher in the *rml1* mutant (Fig. 3B, D). These data revealed that the *rml1* mutant exhibited reduced cell size in conjunction with increased cell number, but overall the result was a smaller plant.

### A mutation in a the *Ribosomal Protein L3* gene causes the *minute*-*like rml1* phenotypes

**Fig. 4.**
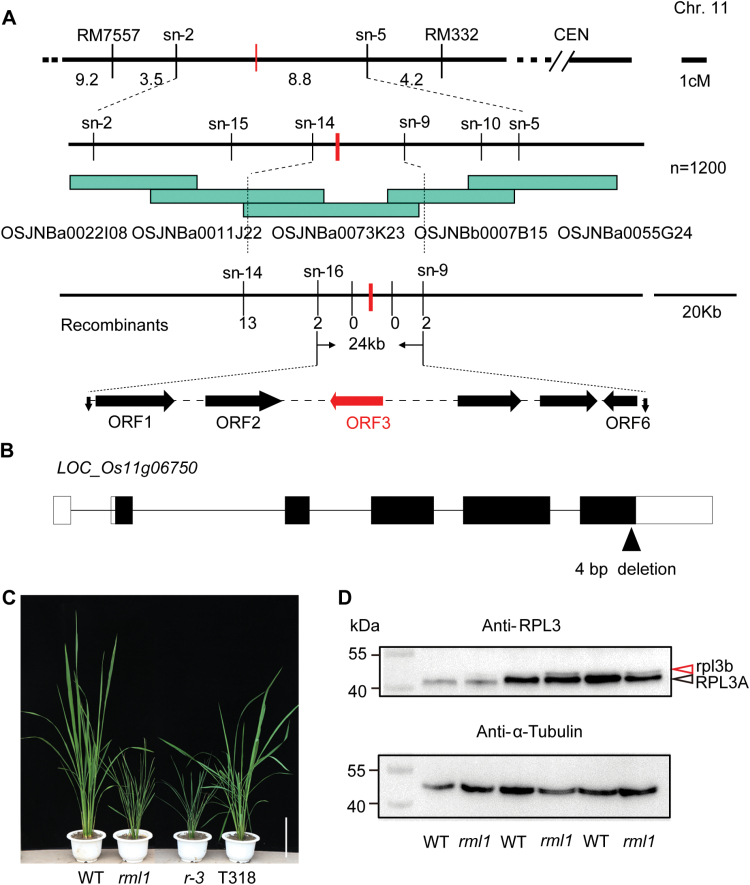
Map-based cloning and characterization of the *RML1* gene. (A) Fine mapping of the *RML1* gene on chromosome 11. The *RML1* locus was narrowed to a 24-kb region containing six predicted ORFs. (B) Schematic of the *RML1* gene and mutations in the *rml1* mutant. *rml1* has a 4-bp deletion in the last exon. Lines, open boxes, and black boxes indicate introns, non-transcribed regions, and exons, respectively. (C) Gross morphologies of wild-type, *rml1*, *r-3* (recessive genotype from BC_2_F_3_), and T318 transgenic lines (complemented with a 10.3-kb genomic fragment of *ORF3*) plants. (D) Immunoblot analysis of the OsRPL3 protein in wild-type and *rml1* seedlings. A gradient experiment was conducted with samples loaded in gels (2, 5 and 8 μl). Anti-α-Tubulin antibody was used as a loading control. Black and red arrowheads indicate RPL3A and rpl3b proteins, respectively. Scale bar in (C) is 20cm.

To isolate the *rml1* gene, ten plants with the recessive *rml1* phenotype were selected from the F_2_ progeny of a cross between *rml1* and the *japonica* cultivar 02428. The mutant gene was located in a 16.5-cM interval on the short arm of chromosome 11. We further mapped the *rml1* locus to a 24-kb region between markers sn-16 and sn-9 on the BAC clone OSJNBb0073K23 using a further 1200 F_2_ plants with the recessive phenotype (Fig. 4A; primer sequences are listed in Supplementary Table S1). Six ORFs in this region were predicted by the Rice GAAS database (Rice Genome Automated Annotation System, http://ricegaas.dna.affrc.go.jp; Supplementary Table S2). Sequence analysis revealed that the third ORF (*LOC_Os11g06750*) in *rml1* had a four-base deletion in the last exon, leading to a frame shift and presumably forming a protein 4kDa larger than the wild-type (Fig. 4B; Supplementary Fig. S4). To further confirm whether *ORF3* was the *RML1* allele, a 10.3-kb genomic DNA fragment including the native promoter was transformed into calli derived from a homozygous line of *r-3* backcrossed to the *japonica* cv. Dian Jing You (DJY). Of 21 T_0_ plants generated, 13 independent positive transgenic plants phenocopied the wild-type (Fig. 4C). Therefore, we concluded that the mutation of *ORF3* was responsible for *rml1*. The *ORF3* encodes a putative ortholog of yeast Ribosomal L3 protein, RPL3B, and consists of 389 amino acids. Another highly homologous protein (RPL3A) with almost the same molecular size also occurs in rice. We next used a specific antibody against OsRPL3 to confirm that the mutation caused the redundant protein. Only one thick band was detected in the wild-type, whereas *rml1* had two bands (Fig. 4D). Given the molecular size of the RPL3 proteins, we believe that the upper band in *rml1* is the mutant rpl3b protein and the lower band is the RPL3A protein.

### Expression pattern and subcellular localization of RML1

**Fig. 5.**
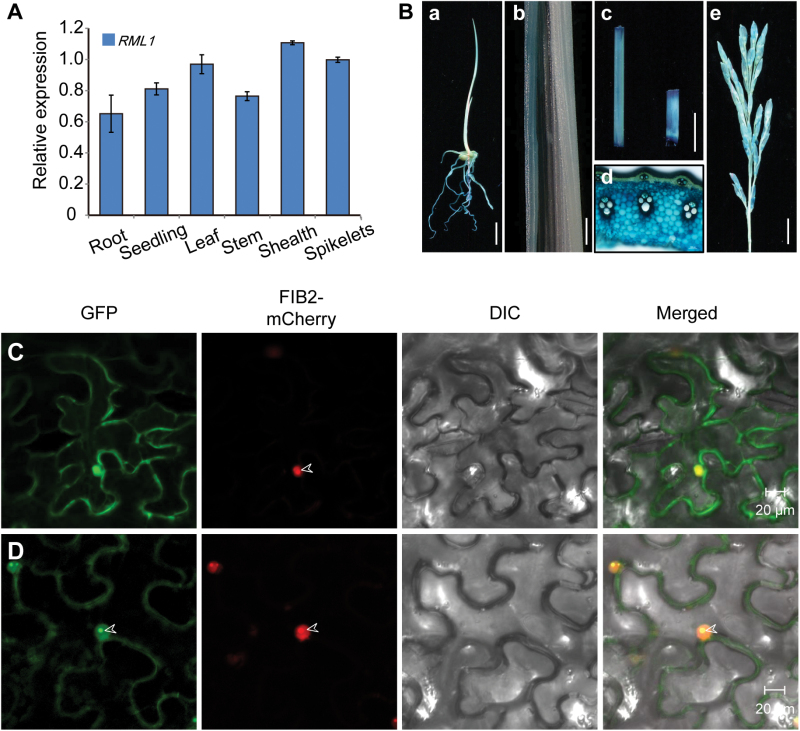
Expression pattern of *RML1* and the subcellular location of RML1. (A) Real-time PCR data showing that *RML1* is expressed in various tissues. (B) GUS staining of various tissues in the *pRML1*: *GUS* transgenic lines: root (a); leaf (b); stem and sheath (c); stem cross- section (d); spikelet (e). (C) Subcellular localization of the GFP protein in *Nicotiana benthamiana* epidermal cells. Free GFP signals were located in the cytoplasm. (D) RML1-GFP fusion protein was localized to the nucleoplasm and cytoplasm and concentrated in the nucleolus. FIB2-mCherry was specifically localized to the nucleolus as a marker. Arrowheads indicate the nucleolus. Scale bars: 10mm (B-a, c); 1mm (B-b); 100 μm (B-d); 20 μm (C, D). *Ubiquitin* (*UBQ*) was used as an internal control in the real-time PCR analyses. Error bars indicate ±SD (*n*=3).

Expression analysis revealed that *RML1* was expressed in all organs tested, including roots, leaves, leaf sheaths, stems, and spikelets (Fig. 5A). The expression levels of *RPL3B* were higher in leaves, sheaths, and spikelets than in roots and stems. To further confirm the expression profile of *RML1*, a *β*-*glucuronidase* (*GUS*) gene driven by the *RML1* promoter (approximately 1.8kb upstream of the translation start site) was transformed into calli of cv. Nipponbare. GUS activity was detected in young roots, seedlings, young leaf blades, leaf sheaths, stems, and panicles, and this was consistent with results from real time-PCR (Fig. 5B). The ubiquitous expression of *RML1* suggested that it might have a pleiotropic role in rice plant development.

BLASTP (http://blast.ncbi.nlm.nih.gov/Blast.cgi) found that RML1 was predicted to be a Ribosomal L3 superfamily protein. During the process of ribosome biogenesis, ribosomal proteins are synthesized in the cytoplasm and then imported into the nucleolus to participate in ribosome subunit assembly, and ribosomes that have translation ability are present in the cytoplasm (Byrne, 2009). To determine the subcellular localization of RML1, GFP was fused to the C-terminus of RML1 and the fusion gene was transiently expressed in leaf epidermal cells of *Nicotiana benthamiana*. RML1-GFP protein was localized to the nucleoplasm and cytoplasm, and concentrated in the nucleolus (Fig. 5C, D). Thus, RML1 is mainly localized in the cytosol as well as in the nucleus.

### *RPL3A* and *RPL3B* are differentially expressed

**Fig. 6.**
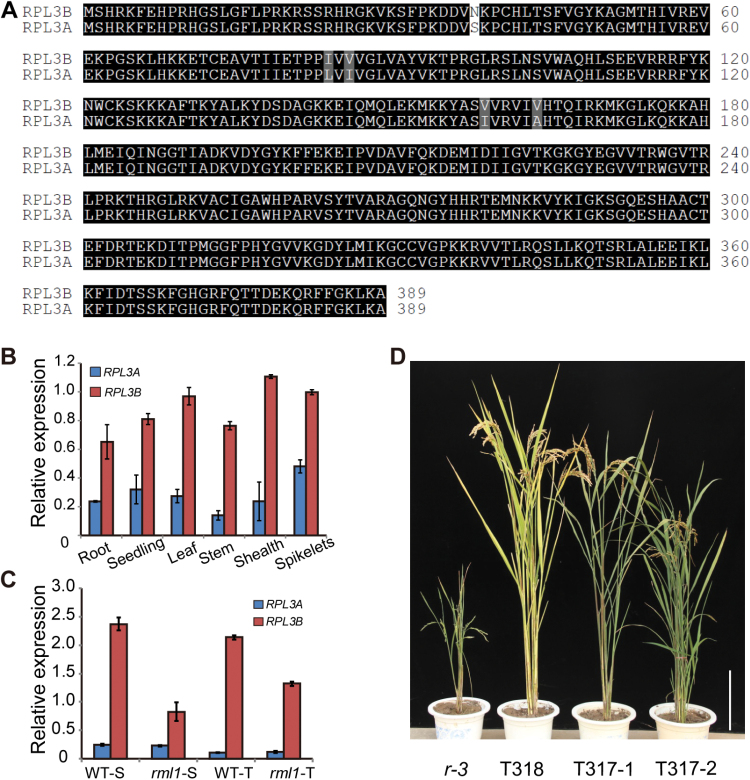
Function comparisons between RPL3A and RPL3B. (A) Clustal alignment of the two rice RPL3 amino acid sequences (RPL3A and RPL3B). Identical and similar residues are shaded black and grey, respectively; the difference is highlighted with no shading. (B) Expression of *RPL3B* was higher than *RPL3A* in all tissues analysed. (C) Results from real-time PCR assay showing that expression of the *RPL3B* gene is reduced in the *rml1* mutant, whereas that of *RPL3A* is unchanged relative to the wild-type. (D) Genetic complementation of *rml1* with *pRML1*:*gRML1* (T318) and *pRML1*:*cRPL3A* (T317) constructs. *Ubiquitin* (*UBQ*) was used as an internal control in real-time PCR. Error bars indicate ±SD (*n* 3). Abbreviations: S, seedling stage; T, tilling stage. Scale bar in (D) is 20cm.

The two RPL3 family members, *RPL3A* and *RPL3B*, in rice share 89.9% identity at the transcript level and 98.7% identity at the amino acid level (Fig. 6A). Real-time-PCR showed that *RPL3B* is more abundant than *RPL3A* in all tissues (Fig. 6B). Previous studies have shown that genetic defects in individual ribosomal components cause deleterious developmental effects in a gene dosage-dependent manner (Ito *et al.*, 2000; Rosado *et al.*, 2010). To determine whether *RPL3A* provides dosage compensation for *rpl3b*, we analysed their expression levels. In the *rml1* mutant, the expression of *RPL3B* was greatly decreased, but expression of *RPL3A* was unchanged (Fig. 6C), suggesting that *RPL3A* cannot compensate for the mutation of *RPL3B*. In addition, we fused the cDNA of *RPL3A* to the promoter of *RPL3B* and transformed this plasmid into the calli of the mutant. Transgenic lines T317 rescued the mutant phenotypes, despite a slight delay in plant growth compared to T318 lines (*RPL3B* promoter: *RPL3B* genomic DNA) (Fig. 6D; Supplementary Fig. S5A). The small difference in complementation efficiency might result from lower expression of *RPL3A* under control of the 3B promoter in T317 compared to *RPL3B* in T318 lines (see Supplementary Fig. S5C). Thus, the different expression levels of *RPL3A* and *RPL3B* could be responsible for the distinct functional difference in plant development.

### The *rml1* mutant has altered ribosomal structure

**Fig. 7.**
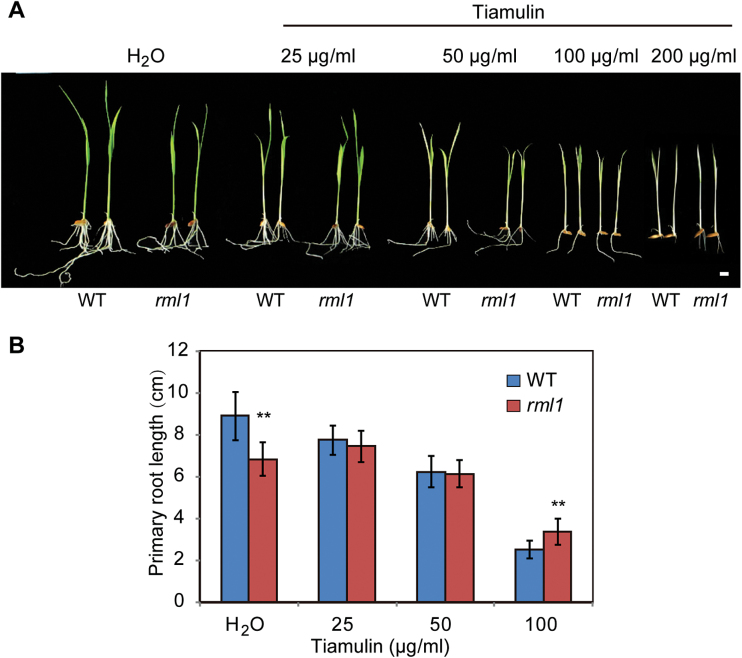
The *rml1* mutant shows slight resistance to tiamulin antibiotic. (A) Wild-type and *rml1* mutant plants treated with different concentrations of tiamulin antibiotic. Wild-type and *rml1* seeds were surface-sterilized and germinated on agar plates with or without the antibiotic. (B) Lengths of primary roots were measured after 7 d. Error bars indicate ±SD (*n*=10). Student’s *t*-test was used for statistical analysis (*, *P*<0.05; **, *P*<0.01).

RPL3 is one of only two proteins capable of initiating *in vitro* assembly of *E. coli* large ribosomal subunits (Nowotny and Nierhaus, 1982), and it is one of the few essential proteins for peptidyltransferase activity (Schulze and Nierhaus, 1982). We hypothesized that the mutant rpl3b ribosomal protein might have undergone a structural change in the *rml1* mutant. In *E. coli*, mutation of *RPL3* increases its tiamulin resistance by alteration of the drug-binding site at the peptidyl transferase centre, probably by its effect on the rRNA structure responsible for the tiamulin resistance (Bosling *et al.*, 2003; Klitgaard *et al.*, 2015). We therefore analysed the responses of wild-type and *rml1* plants treated with a concentration series of tiamulin by measuring the lengths of primary roots, and found that the *rml1* mutant displayed mild resistance to tiamulin compared to the wild-type (Fig. 7). Treatment with other antibiotics that target different ribosomal locations in prokaryotes resulted in no obvious changes between the wild-type and *rml1* (Rosado *et al.*, 2010; Supplementary Fig. S6). These results suggest that mutation of *RPL3B* probably causes a structural alteration in the peptidyltransferase centre.

### Defective ribosome biogenesis alters the ribosome profile in *rml1*

**Fig. 8.**
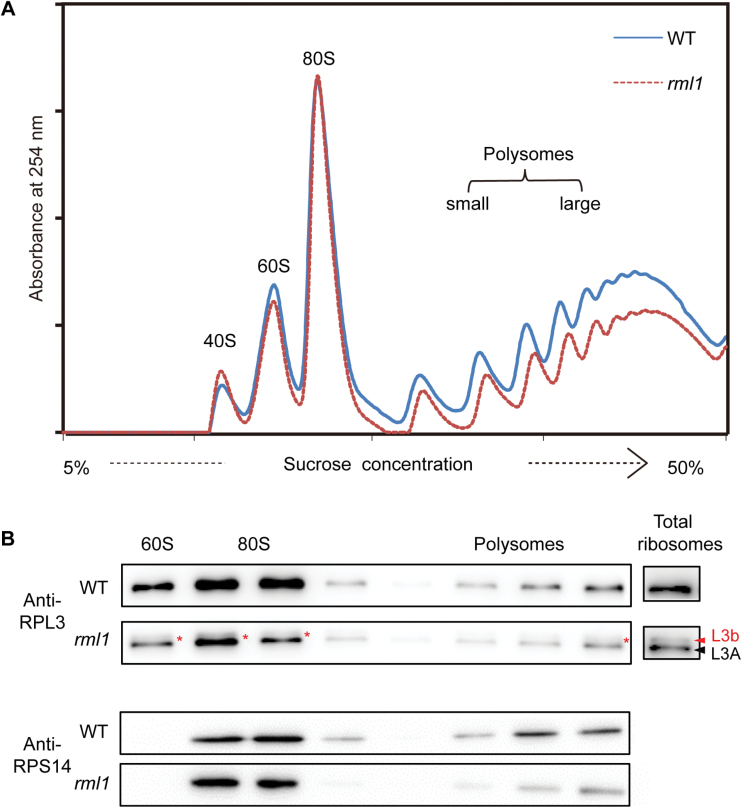
Absorbance profiles of ribosomes at 254nm and immunoblot analysis of the OsRPL3 protein in ribosomal fractions. (A) Ribosome extracts from wild-type and *rml1*seedlings were fractionated on a 5–50% sucrose density gradient. Absorbamce peaks at 254nm representing free 40S and 60S subunits, 80S monosomes, and polysomes are indicated. (B) Fractions from the gradient shown in (A) were collected and subjected to immunoblotting with the indicated antibodies. HsRPS14 was used as the marker for the 40S subunit. Red and black arrowheads indicate rpl3b and RPL3A, respectively. Red asterisks indicate rpl3b. All assays were performed at least three times.

To investigate a potential role of RPL3B in ribosomal biogenesis, we performed ribosomal profiling in wild-type and *rml1* plants. Total ribosome particles were isolated from cell extracts and centrifuged on a sucrose cushion overnight. The re-suspended polysomes were fractionated by sucrose density gradient ultracentrifugation and measured at 254nm using a UV detector. The *rml1* mutant showed a clear deficit of the free 60S ribosomal subunit fraction compared to the wild-type, while the accumulation of 40S small subunits was slightly increased (Fig. 8A). The profile for 80S subunits (monosomes) was similar to the wild-type. Significantly reduced accumulation of polysomes in *rml1* plants indicated that the mutant might be mildly defective in translation initiation or have repressed global protein translation activity (Fig. 8A; Garcia-Gomez *et al.*, 2014). The protein compositions of the gradient fractions were subsequently determined by protein gel blotting and the ribosomal small protein 14 (HsRPS14) was used as a marker to monitor sedimentation of 60S and 40S ribosomes (Fig. 8B; Supplementary Fig. S7). In *rml1* plants, the rpl3b protein also occurred in 60S and 80S subunits and polysomes, suggesting that the mutant rpl3b protein also participated in ribosome biogenesis. Both normal and mutant types of RPL3B also bound 25S rRNA *in vitro* (see Supplementary Fig. S8A).

Real-time PCR analyses to detect whether the mutation of RPL3B resulted in defects in pre-rRNA processing or rRNA accumulation showed that 35S pre-RNA, 25S rRNA, and 18S rRNA were slightly increased in the *rml1* mutant (Supplementary Fig. S8B). These phenotypes have also been observed in a yeast *rpl3* [I282T] mutant that showed a similar polysome profile to *rml1* (Garcia-Gomez *et al.*, 2014). Our results indicate that depletion of RPL3B in the *rml1* mutant causes defective biogenesis of the 60S ribosome large subunit and polysomes as well as defects in pre-rRNA processing.

## Discussion

### RPL3 family members are involved in rice plant growth and development

Multiple copies of the ribosomal proteins (RPs) in yeast have both redundant and non-redundant roles in function (Deutschbauer *et al.*, 2005; Komili *et al.*, 2007; Dean *et al.*, 2008). Most RP families in Arabidopsis consist of multiple members, ranging from two to seven (Barakat *et al.*, 2001), but some copies are non-expressed pseudogenes (Rosado *et al.*, 2010). RP genes in rice also have multiple copies (Shi *et al.*, 2014), but it remains unclear whether or how the duplicated genes function in plant development. In Arabidopsis, some members of the RP family with different functions have been associated with different expression levels or patterns. *RPL23aA* has a higher expression level than *RPL23aB*, and knock-down of *RPL23aA* leads to various developmental defects, whereas knock-down of the *rpl23aB* mutant does not (Degenhardt and Bonham-Smith, 2008*a*, *b*). In addition, paralogs of *RPL5* and *RPL10* family members also have distinct roles caused by their differential expression patterns (Fujikura *et al.*, 2009; Falcone *et al.*, 2010, 2013).

In this study we report for the first time in rice that RPs have functions in plant growth and development. We identified a *rice minute-like* mutant caused by mutation of *RPL3B*. In rice, *RPL3B* has an expressed paralog, *RPL3A*, and the coding regions as well as amino acid sequences are highly similar. We found that *RPL3A* is expressed at a lower level than *RPL3B* during all periods of plant development; however, the expression level of *RPL3A* in *rml1* plants is unchanged compared to the wild-type (Fig. 6). According to the ribosome heterogeneity model (Horiguchi *et al.*, 2012), the differential expression levels of the two *RPL3* genes might be responsible for their distinct functions in rice plant development, and it was observed that *RPL3A* provides no dosage compensation for the *RPL3B* mutation. Conversely, *RPL3B* is essential for translation of certain specific transcripts during plant development, a function that could not be supplemented by *RPL3A*. The relationship between the two isoforms in rice needs to be further clarified with mutants of *RPL3A*.

### Mutation of *RPL3B* had a deleterious effect on polysome synthesis

Mutation or depletion of RPL3p has been shown to have effects on ribosome structure and function in yeast (Petrov *et al.*, 2004; Meskauskas and Dinman, 2007; Rosado *et al.*, 2007). In this study, the rpl3b protein had a deletion at its C-terminus and formed a protein ~4kDa larger than the wild-type. Ribosomal profile analysis showed that levels of both the 60S subunit and polysomes were decreased. In addition, immunoblot analysis of the fractions indicated that the mutant rpl3b protein was present in 60S, 80S, and polysome particles, although the band was weak (Fig. 8). This indicates that a few rpl3b proteins are assembled into 60S subunits as well. The *rml1* mutant also displayed mild resistance to tiamulin compared to the wild-type (Fig. 7). An EMSA assay proved that the mutant rpl3b protein can also bind the sarcin/ricin RNA domain of 25S rRNA *in vitro* in a similar manner to the wild-type (see Supplementary Fig. S8A) (Uchiumi *et al.*, 1999). These results suggest that the peptidyltransferase centre was slightly changed.

Reduction in the polysome/monosome ratio in yeast is associated with translation initiation defects (Deplazes *et al.*, 2009; Garcia-Gomez *et al.*, 2014; Visweswaraiah *et al.*, 2015). Our polysome profile assays found that polysomes were significantly decreased in the *rml1* mutant, while monosomes were unchanged (Fig. 8). Notably, the polysome/monsome ratio in the *rml1* mutant was reduced relative to the wild-type. One explanation would be that the 60S particles are not well stabilized and a few aberrant 60S r-particles containing the mutated form of RPL3B were lost after assembly, leading to a decrease in the level of 60S and, in turn, to a small excess of 40S subunits. Considering the latest model for ribosome function of Horiguchi *et al.* (2012) together with our results, we deduce that ribosome insufficiency and aberrancy, or defective translation initiation, caused developmental abnormalities in the *rml1* mutant.

### A deficit in ribosomal biogenesis alters auxin-related responses

Our study demonstrated that deletion of *RPL3B* disturbs the vascular patterns in leaves and stems of rice, including reduced numbers of large veins and small veins, and altered midrib morphology and vascular bundle size. In addition, some leaves had no proper blade at their lower sections (Supplementary Fig. S3A). Previous studies have reported that polar auxin transport plays an essential role in formation of the vascular system in Arabidopsis (Scarpella *et al.*, 2006, 2010). We therefore hypothesize that the abnormal vascular patterns in the rice *rml1* mutant might be associated with auxin distribution.

Active auxin in plants is synthesized in areas that are associated with rapidly dividing and growing tissues. The shoot apical meristems and young leaves are considered to be the primary sites of auxin synthesis, and auxin moves from apical to basal regions (basipetally) in fulfilling its essential role in plant development (Ljung *et al.*, 2001). In Arabidopsis, polar localization on the plasma membrane protein PIN1 has been proposed as the auxin efflux carrier (Palme and Galweiler, 1999). Recent reports have shown that auxin displays a key role in both initiation and elaboration of final morphology of both leaves and vascular networks (Scarpella *et al.*, 2010). Some rice mutations that display vascular defects have also been associated with auxin transport (Qi *et al.*, 2008; Cho *et al.*, 2013; Fujita *et al.*, 2013).

We found that the free contents of IAA in *rml1* were higher than in the wild-type (Supplementary Fig. S9A), whereas auxin signal transport and transduction genes, such as *OsPINs* and *auxin response factors* (*ARFs*), were significantly down regulated (Supplementary Fig. S9B, C). Moreover, expression of the *NAL1* gene that is related to leaf vascular and plant architecture development was decreased in the *rml1* mutant (Supplementary Fig. S9B). Reduction in polar auxin transport capacity in the *nal1* mutant has been shown to affect vein patterning (Qi *et al.*, 2008; Fujita *et al.*, 2013). Antisense knock-down of *OsARF1* (*ARF23*) also caused dwarf plants with small curled leaves, defects in reproductive development, and late flowering (Waller *et al.*, 2002), similar phenotypic effects to the *rml1* mutant. We therefore deduce that auxin transport or transduction defects may lead to the abnormal leaf morphology and plant architecture phenotypes displayed in the *rml1* mutant. Alternatively, defects in ribosome biogenesis may disrupt translation of mRNAs that are necessary to guide auxin distribution or transduction in developing leaves.

Individual mutations in RPs (*rpl24*, *rpl4d*, and *rpl5a*) cause specific auxin-related phenotypes in Arabidopsis, and these RPs regulate the translation of the auxin response factors (*ARF3*/*5*) via translation of the upstream opening reading frames at the *ARF*’s 5′ leader sequence (Nishimura *et al.*, 2005; Horiguchi *et al.*, 2012; Rosado *et al.*, 2012). However, functional analyses of rice *ARF* genes have been reported only for *OsARF1* (*ARF23*), *ARF12*, *ARF16*, *ARF19*, *ARF24*, and *ARF25* where most mutants have defects in root growth and nutritional uptake (Waller *et al.*, 2002; Li *et al.*, 2014; Shen *et al.*, 2015; Zhang *et al.*, 2015). Further studies are needed to clarify the relationship between ribosomal proteins and *ARF*s, and to demonstrate whether a similar mechanism occurs in ribosomal protein-mediated translation of *ARF*s in rice.

## Supplementary Data

Supplementary data are available at *JXB* online.

**Figure S1.** Gross morphology of wild-type (93-11), *rml1*, and heterozygous (F_1_) plants.

**Figure S2.** Phenotypic analyses of the wild-type and the *rml1* mutant.

**Figure S3.** Phenotypes of leaf blades of wild-type and *rml1* plants.

**Figure S4.** Analysis of the amino acid sequences and protein molecular weight between RPL3B and rpl3b.

**Figure S5.** Transgenic complementation of *rml1*.

**Figure S6.** Antibiotics resistance assays.

**Figure S7.** Fractions from the gradient for immunoblotting analysis.

**Figure S8.** EMSA assays and accumulation of pre-rRNA precursors.

**Figure S9.** Auxin content and expression of auxin-related genes in wild-type and *rml1* plants.

**Table S1.** Primers used for mapping.

**Table S2.** Six candidate ORFs in the 24-kb region.

**Table S3.** Primers used for vector construction.

**Table S4.** Primers used for real-time PCR analysis.

Supplementary Data
