# Supplementary material for: The RICE MINUTE-LIKE1 (RML1) gene, encoding a ribosomal large subunit protein L3B, regulates leaf morphology and plant architecture in rice
Source: J Exp Bot. 2016 May 28;67(11):3457–69. doi: 10.1093/jxb/erw167 (PMC4939763; doi:10.1093/jxb/erw167)
Supplement: Supplementary Data [file supp_67_11_3457__index.html]

The RICE MINUTE-LIKE1 (RML1) gene, encoding a ribosomal large subunit protein L3B, regulates leaf morphology and plant architecture in rice — Supplementary Data 

# The *RICE MINUTE-LIKE1* (*RML1*) gene, encoding a ribosomal large subunit protein L3B, regulates leaf morphology and plant architecture in rice

## Supplementary Data

Data files

- supplementary\_tables\_S1\_S4\_figures\_S1\_S9.pdf - Supplementary Data
