# Supplementary material for: The RICE MINUTE-LIKE1 (RML1) gene, encoding a ribosomal large subunit protein L3B, regulates leaf morphology and plant architecture in rice
Source: J Exp Bot. 2016 May 28;67(11):3457–69. doi: 10.1093/jxb/erw167 (PMC4939763; doi:10.1093/jxb/erw167)
Supplement: Supplementary Data [file supp_erw167_supplementary_tables_S1_S4_figures_S1_S9.pdf]

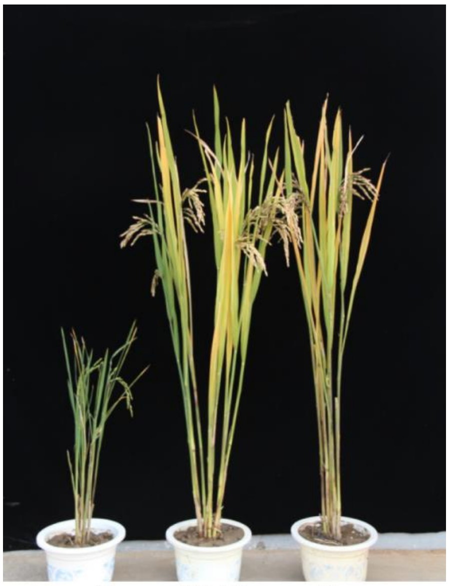

Fig. S1. Gross morphologies of wild-type (93-11), *rml1* and heterozygous (F_1) plants.

Plant architechture of the F_1 is the same as WT (93-11).

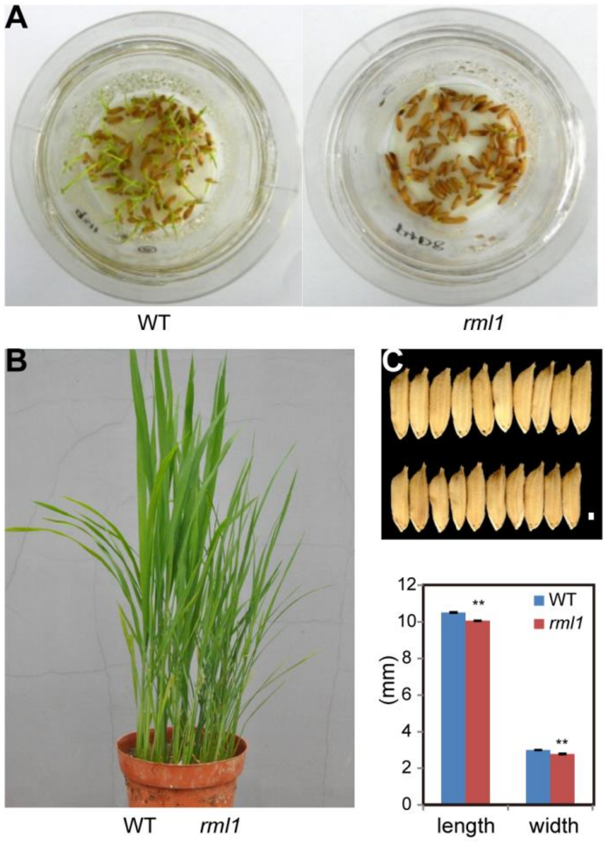

Fig. S2. Phenotypic analyses of wild-type and *rml1* mutant.

(A) Comparsion of wild-type (WT) and *rml1* mutant at 3 days after germination. (B) Seedlings of wild-type (WT) (left) and *rml1* (right) mutant at one month. (C) Comparison of wild-type (WT) and *rml1* mutant seeds. Bar, 1 mm. Student's t-test was used for statistical analysis (*, P<0.05; **, P < 0.01).

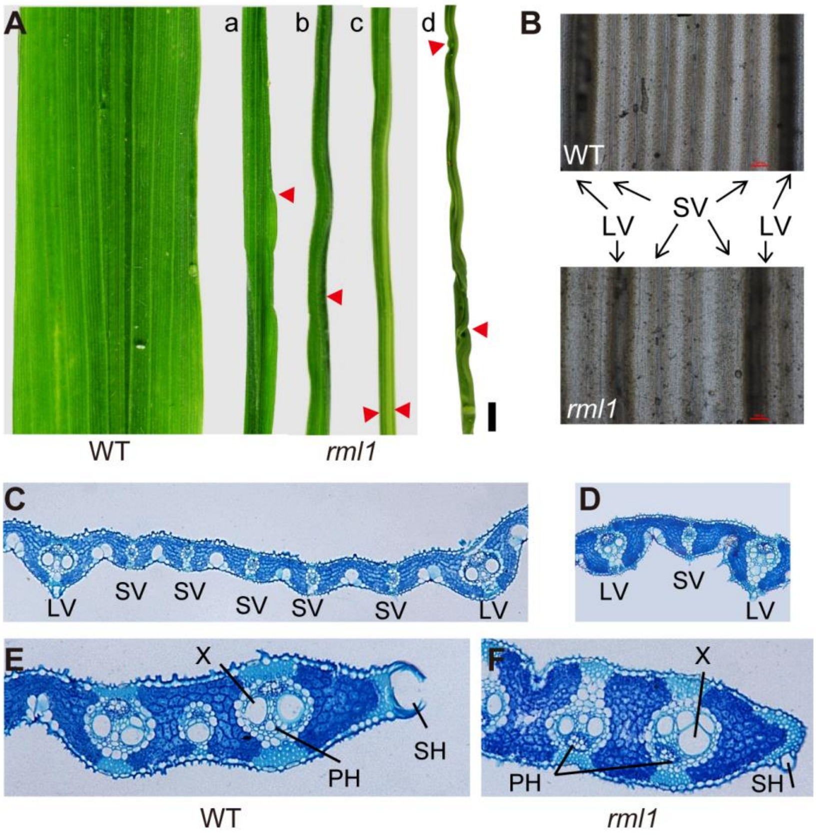

Fig. S3. Phenotypes of leaf blades of wild-type and *rml1* plants.

(A) Abnormal leaf morphology of *rml1* mutant. Various phenotypes of *rml1* leaves (a-d). Red arrowheads indicate deformed leaf blades. (B) Whole-mount clearing of the mature flag leaves of wild-type and *rml1* plants. (C-F) Transverse sections of the middle parts of flag leaf blades of wild-type and *rml1* stained with toluidine blue. LV, large vascular bundle; PH, phloem; SH, sawtooth hair; SV, small vascular bundle; X, xylem.

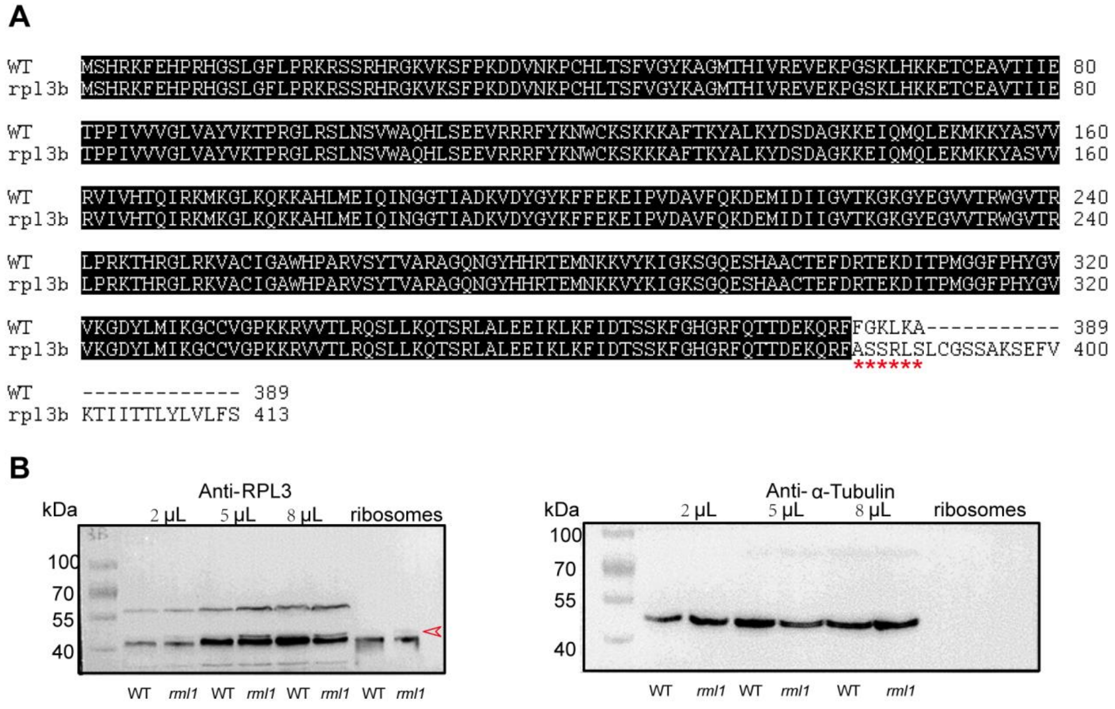

Fig. S4. Analysis of the amino acid sequences and protein molecular weights of RPL3B and rpl3b.

(A) Clustal alignment of the RPL3B and rpl3b amino acid sequences. Red asterisks indicated altered amino acids. (B) Immunoblot analysis of OsRPL3 protein in wild-type and *rml1* seedlings and ribosomes. A gradient experiment of samples in gels (2, 5 and 8μL). Anti-α-Tubulin antibody was used as the loading control. Arrowhead indicates rpl3b.

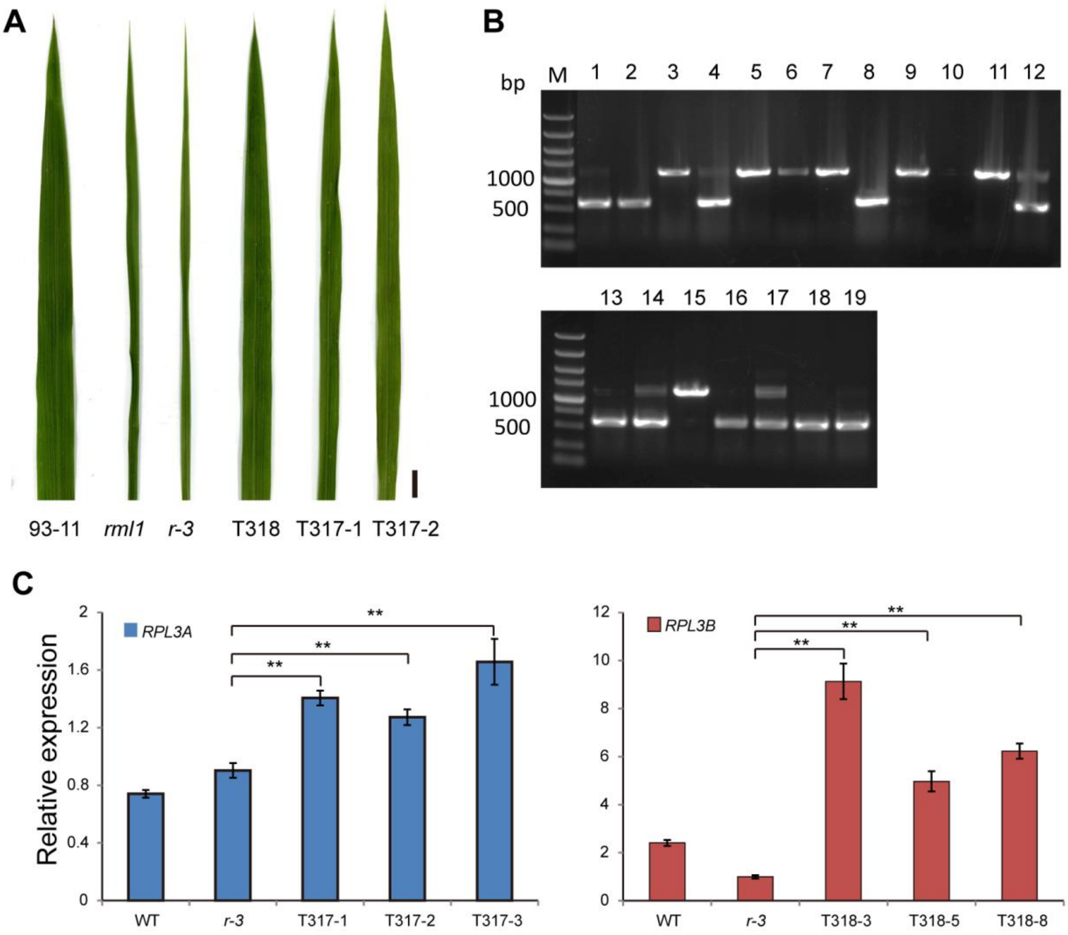

Fig. S5. Transgenic complementation of *rml1*.

(A) Leaf morphologies of wild-type (93-11), *rml1*, *r-3*, T318 (transgenic line) and T317 (transgenic line) plants. Bar, 1 cm. (B) Molecular identification in T317 transgenic lines. PCR products containing the ~500bp band indicate the positive lines. (C) Expression levels of *RPL3A* in transgenic lines T317 and *RPL3B* in T318 seedlings. Student's t-test was used for statistical analysis (*, $P<0.05$; **, $P < 0.01$). *Ubiquitin* (*UBQ*) was used as an internal control in real-time PCR analyses. Error bars indicate ±SD (n = 3).

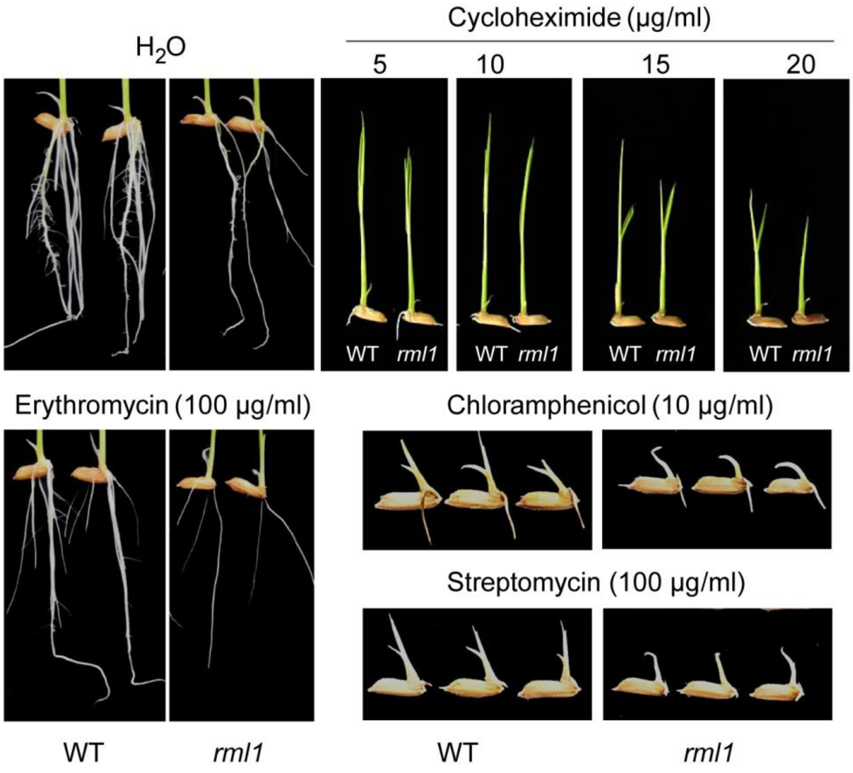

Fig. S6. Antibiotic resistance assays.

Wild type and *rml1* mutant were treated with different concentrations of various antibiotics. Wild-type and *rml1* seeds were surface sterilized and directly germinated on agar plates with or without antibiotic.

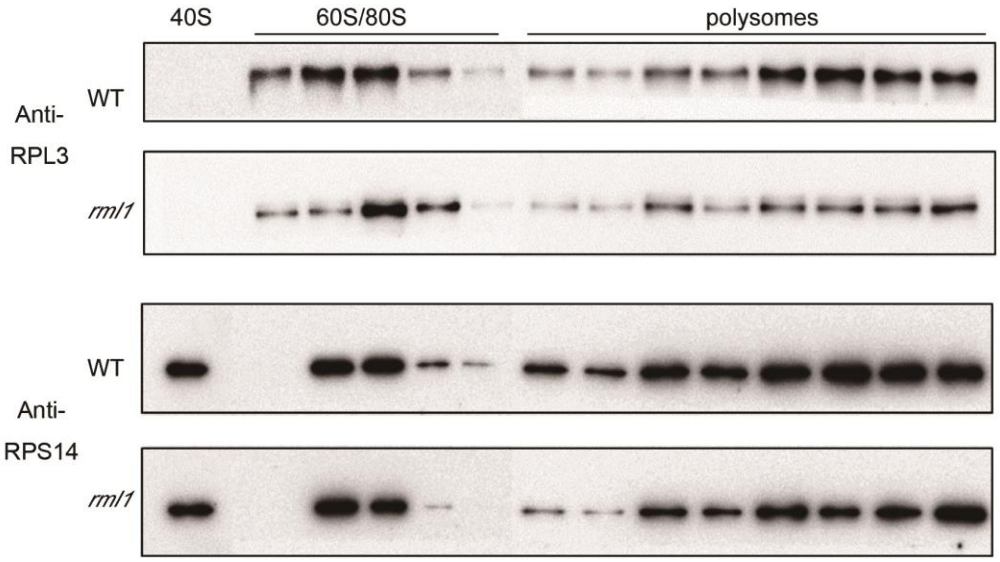

Fig. S7. Gradient fractions for immunoblotting analysis.

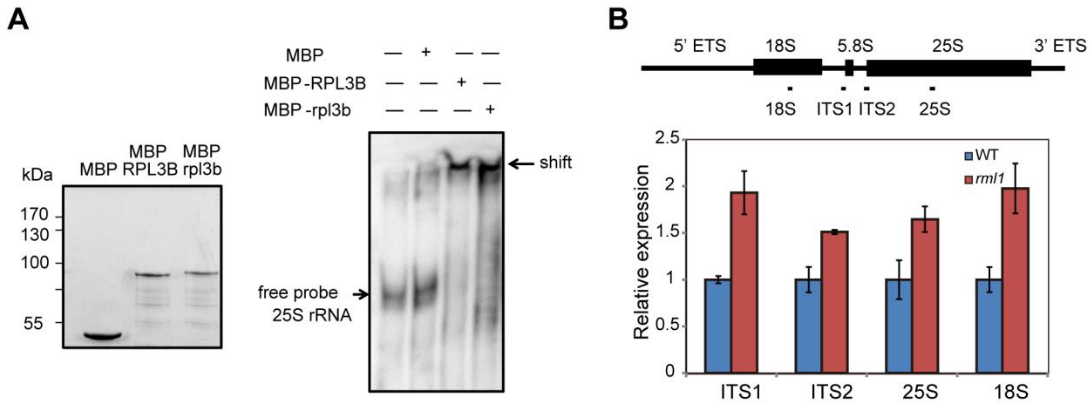

Fig. S8. EMSA assays and accumulation of pre-rRNA precursors.

(A) EMSA assays of RPL3B and rpl3b proteins and sequences of 25S rRNA. (B) Diagram illustrating the 35S pre-rRNA transcript and PCR-amplified regions. Real-time RT-PCR analyses of 25S, 18S, and rRNA precursors (ITS1 and ITS2). *Ubiquitin* (*UBQ*) was used as an internal control in real-time PCR. Error bars indicate ± SD (n = 3). Student's t-test was used for statistical analysis (*, P < 0.05; **, P < 0.01.)

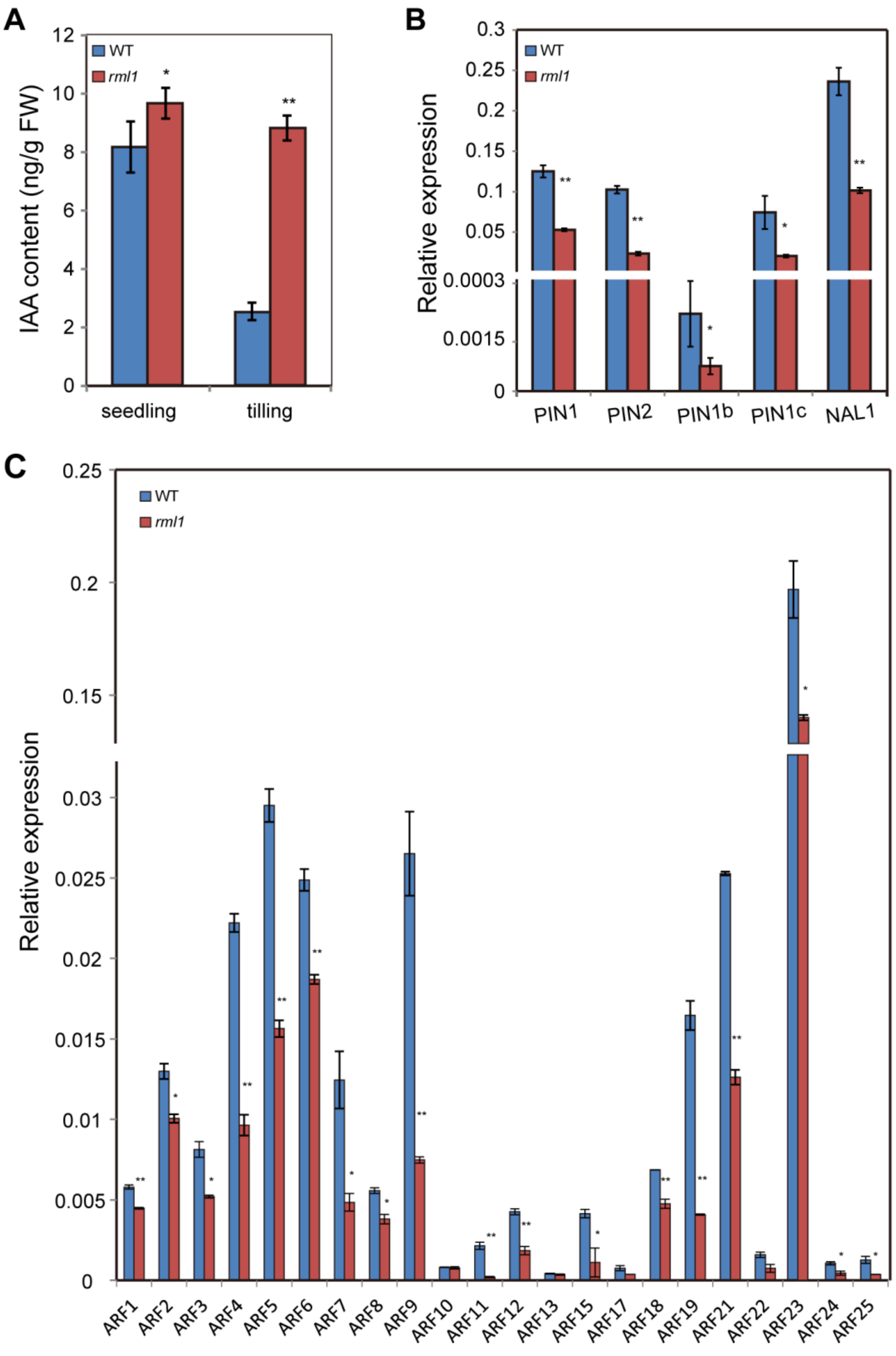

Fig. S9. Auxin content and expression of auxin-related genes in wild-type and *rml1* plants.

(A) Endogenous free-IAA concentrations in leaf tips of wild-type (WT) and *rml1* at the seedling and tilling stages using liquid chromatography mass spectrometry (LC-MS). FW, fresh weight. (B) Expression analysis of *PINs* and *NAL1* in wild-type and *rml1* leaves. (C) The expression of ARFs in wild-type and *rml1* plants. Error bars indicate ± SD (n=3). Student's t-test was used for statistical analysis (*, $P < 0.05$; **, $P < 0.01$.)

Table S1. Primers used for mapping

Primer name	Forward sequence	Reverse sequence
RM7557	GTGTACTGCCATGAAAGGCC	GAAGTGCCTTTGCAGGAGAG
RM332	GCGAAGGCGAAGGTGAAG	CATGAGTGATCTCACTCACCC
sn-2	GGGAAGCGTTTAAGTGGTG	AAAGCCTACATGGAAGAGTGA
sn-5	CCCTCGGTAACTGTAAGCTCCTCA	GCAACCTGCTGCCATCTCCTCT
sn-9	TTCAACATAGTTTTCTTTTCAGCTT	ACAGAGTATTATTCTTGGCACGG
sn-10	GCTCTTGTTCTTATGCTTAC	ATGTGAATTATGTTGGATGA
sn-14	ATGCCTAATAGGTCGAATTTCTGA	CCTTCCTCTTTCTCGGTCATTT
sn-15	CCGTAAGAGTGAACGTGGAG	CGACCGTGTAGGGTATGAGA
sn-16	TGTTCGTCGTCGCTGAGTTA	AGATCGTCCTGATGGGCTTC

Table S2. Six candidate ORFs in 24 kb region

ORF	Gene annotation
ORF1	putative Solution Structure Of Archaeon Dna-Binding Protein Ssh10b
ORF2	putative RecName: Full=Pre-mRNA-splicing factor cwc-22
ORF3	putative RecName: Full=60S ribosomal protein L3
ORF4	putative Syntaxin 6
ORF5	putative ECA1 protein
0RF6	hypothetical protein

The candidate ORF3 is highlighed in red.

Table S3. Primers used for vector construction

Primer name	Forward sequence	Reverse sequence	Used for
1381p	TGAAGCTTTAGGCGACAGGATGAGGAA	AACTGCAGAATACAGCACCGCACAAGC	GUS assay
J2pgt	AACTGCAGCGTGGATCTGATGGAGTTTGC	TGGAGCTCTCCGCTTCGTCTATGCAACTC	complementation
J2BpAc	AACTGCAGATGTCGCACAGGAAGTTCGAG	CGAGCTCCTAGGCCTTGAGCTTGCCGAAGA	complementation
MBP-RPL3B	CGGAATTCATGTCGCACAGGAAGTTC	CCCAAGCTTCTAAGCCTTGAGCTTGCC	EMSA
MBP-rpl3b	CGGAATTCATGTCGCACAGGAAGTTC	CCCAAGCTTTCAAGAGAAGAGAACCAA	EMSA
probe25S rRNA	CGTGCCGCGATAGTAATTCAA	GGCGCCGCTTGCTAGCTTGGAT	EMSA
1305-RPL3B-GFP	GTCCGGAGCTAGCTCTAGAATGT CGCACAGGAAGTTC	GCCCTTGCTCACCATGGATCCAGC CTTGAGCTTGCCGAA	Subcellular localization
FIB2-mcherry	GCTCTAGAATGAGACCTCCTCTAAC	CGGGATCCAGCAGCAGTAGCAGC	Subcellular localization
T317	AAACGAAGAGGTCAAGTGGAGGTA	GTGTCATTCCAGCCTTGTAGCC	Molecular identification

Sequences marked in red represent the restriction site.

Table S4. Primers used for real-time PCR analysis

Primer name	Gene ID	Forward sequence	Reverse sequence
UBQ	LOC_Os03g13170	CACCCTGGCTGACTACAACA	TTCTTCTTGCGGCAGTTGAC
RPL3A	LOC_Os12g07010	TCAGGTAAGGAGGAGGAAGATGTC	TTGTAGCCAACGAAGGAAGTAAGG
RPL3B	LOC_Os11g06750	GACCACCGACGAGAAACAGAGG	GACAGCAGAAGGGAGCACAAATTC
ITS1	AF169230.1	TCAAGGAACACAGCGATACG	AGATATCCGTTGCCGAGAGT
ITS2	AF169230.1	GCTCACGCTGGCTCTAGG	GTAGTCCCGCCTGACCTG
ARF1	LOC_Os01g13520	CCCCAAGAGATCCAAAGCCATC	GCTAGATCCACAGCTCTGCCCA
ARF2	LOC_Os01g48060	AAGAAGGCGTACTGGAATAA	ATCCCATTTCACCACTAAAC
ARF3	LOC_Os01g54990	AATGAAGCGATTTTCAAAGC	CGTACTCAGAAGCAGTAGCC
ARF4	LOC_Os01g70270	CTGCTTTGCCGTGTCCTCAAC	CTTCTCCACCGCCATCTCATT
ARF5	LOC_Os02g04810	GATCACCATCCAAGCTCATCT	GCTGGAAACTGGTCTACAAGG
ARF6	LOC_Os02g06910	TTCCTCCGCTGGATTTCACTC	TACAAAGACGCTCCAACCCGT
ARF7	LOC_Os02g35140	GGTCAATGTAGAACTTAGGGC	ATGTGGCTCAGGTTTTGGGCT
ARF8	LOC_Os02g41800	GAGATGTTCTGCATCGACAG	TCGGTTAGTATGGTAAGCCT
ARF9	LOC_Os04g36054	GGCGGTTTCTCTGTTCTTAGGA	AGATGTGACGGAAATGCCACTC
ARF10	LOC_Os04g43910	GGCGGGAGGAGGAGAATAGTAG	CTGTAGTCCAGCTCCGGGAAGA
ARF11	LOC_Os04g56850	TCTCAGTTGCTGTGTCAAGTTC	TGGAATAGGGAATACATCCGTT
ARF12	LOC_Os04g57610	GACGAGGTGTACGCGCAGATGA	GTGTCGCTCGCCGTCAATGTCT
ARF13	LOC_Os04g59430	GATCTTCAGATTTTAGTTCCCC	CTGTATGGTCTTCATTCCGTCA
ARF15	LOC_Os05g48870	TCCAGCAAGAGAAGAACTAGA	AGTTGAAATATCAATCACCCT
ARF17	LOC_Os06g46410	GGCTGGAGCGTCTTTGTAAGT	GGCATAACAGTTTGTGGGCGG
ARF18	LOC_Os06g47150	ACCCCATTAGATGGCCTAATTCA	GGCTCACCCTCTTCACATTCTGT
ARF19	LOC_Os06g48950	CTTCTGCATAACGTCACTTTAC	TTCTGTCTGTGGTCTCGCTTGT
ARF21	LOC_Os08g40900	CAACCTACGAACCTCTCATCA	TCACAAATTCGGAAGTACTGG
ARF22	LOC_Os10g33940	TCACATTCTGCAACAGGTCC	GCTTTTGAGACAGAGGATTC
ARF23	LOC_Os11g32110	GAAGGAGTGGATGGTTGTCTA	ACCTCTTCTCTTGTGTAGATG
ARF24	LOC_Os12g29520	AGCTCAAGGCTGAGCCTGATAC	TCTTTGGACACATTCCCGTTAC
ARF25	LOC_Os12g41950	ACAGATGAAGTCTATGCTCAG	GCAGAAGTAATTAGTGGGTTG

Analysis of endogenous IAA content

For IAA measurements, samples were extracted, purified and analyzed following the standard procedure for Liquid chromatography mass spectrometry (LC-MS) assays as previously described (Liu *et al.*, 2013). Each sample was analyzed in triplicate.

EMSA

Full length cDNA of *RPL3B* and *rpl3b* were cloned into the pMAL-C2x vector (NEB) to construct the fusion proteins MBP-RPL3B/rpl3b, and the 25S rRNA sequence was cloned into the peasy-T3 vector to transcribe the 25S rRNA probe using T7 RNA polymerase (Roche). The EMSA binding buffer contained 40mM Tris-HCl (pH 7.5), 100 mM Nacl, 0.1 mg/mL BSA, 4 mM DTT, 0.5 mg/mL heparin and 5 units RNasin. After incubation for 30 min at 25℃, the samples were loading on a 6% Tris-borate gel in 0.25×Tris-borate buffer and then transformed to nylon membrane (Millipore). The membrane was incubated with anti-DIG at 1:1000 dilutions, and detected using a CDP-star (Roche).

Liu S, Chen W, Qu L, Gai Y, Jiang X. 2013. Simultaneous determination of 24 of more acidic and alkaline phytohormones in femtomole quantities of plant tissues by high-performance liguid chromatography-electrospray ionization ion trap mass spectrometry. *Analytical and Bioanalytical Chemistry* **405**, 1257-1266.
